# Untangling hidden nutrient dynamics: rapid ammonium cycling and single-cell ammonium assimilation in marine plankton communities

**DOI:** 10.1038/s41396-019-0386-z

**Published:** 2019-03-25

**Authors:** Isabell Klawonn, Stefano Bonaglia, Martin J. Whitehouse, Sten Littmann, Daniela Tienken, Marcel M. M. Kuypers, Volker Brüchert, Helle Ploug

**Affiliations:** 1 Department of Ecology, Environment and Plant Sciences, Stockholm University, 10691 Stockholm, Sweden; 2 Department of Experimental Limnology, IGB-Leibniz-Institute of Freshwater Ecology and Inland Fisheries, 12587 Berlin, Germany; 3 Department of Geological Sciences, Stockholm University, 10691 Stockholm, Sweden; 4 Swedish Museum of Natural History, 11418 Stockholm, Sweden; 5 Max Planck Institute for Marine Microbiology, 29357 Bremen, Germany; 6 Department of Marine Sciences, University of Gothenburg, 40530 Gothenburg, Sweden

**Keywords:** Biogeochemistry, Environmental sciences

## Abstract

Ammonium is a central nutrient in aquatic systems. Yet, cell-specific ammonium assimilation among diverse functional plankton is poorly documented in field communities. Combining stable-isotope incubations (^15^N-ammonium, ^15^N_2_ and ^13^C-bicarbonate) with secondary-ion mass spectrometry, we quantified bulk ammonium dynamics, N_2_-fixation and carbon (C) fixation, as well as single-cell ammonium assimilation and C-fixation within plankton communities in nitrogen (N)-depleted surface waters during summer in the Baltic Sea. Ammonium production resulted from regenerated (≥91%) and new production (N_2_-fixation, ≤9%), supporting primary production by 78–97 and 2–16%, respectively. Ammonium was produced and consumed at balanced rates, and rapidly recycled within 1 h, as shown previously, facilitating an efficient ammonium transfer within plankton communities. N_2_-fixing cyanobacteria poorly assimilated ammonium, whereas heterotrophic bacteria and picocyanobacteria accounted for its highest consumption (~20 and ~20–40%, respectively). Surprisingly, ammonium assimilation and C-fixation were similarly fast for picocyanobacteria (non-N_2_-fixing *Synechococcus*) and large diatoms (*Chaetoceros*). Yet, the population biomass was high for *Synechococcus* but low for *Chaetoceros*. Hence, autotrophic picocyanobacteria and heterotrophic bacteria, with their high single-cell assimilation rates and dominating population biomass, competed for the same nutrient source and drove rapid ammonium dynamics in N-depleted marine waters.

## Introduction

In various aquatic environments, ranging from inland lakes, brackish seas to the global ocean, primary production is fuelled by N_2_-fixation [1] and regenerated nitrogen (N), including ammonium [2, 3]. Only few microorganisms, e.g., filamentous cyanobacteria are able to reduce N_2_ to ammonium [1]. In contrast, ammonium is highly bioavailable and thus the predominant N-compound assimilated by bacterioplankton and phytoplankton [4, 5]. Its cycling is complex, driven by various sources and sinks in plankton communities. In brief, ammonium is consumed through assimilation and nitrification (oxidation of ammonium to nitrite/nitrate). In turn, it is regenerated by bacterial remineralisation of organic N, zooplankton grazing, parasitic infections, or cell lysis [6]. New sources include ammonium leakage from diazotrophic cyanobacteria [7–9] which fix N in excess relative to their cellular C:N ratio.

Ammonium assimilation by individual microbes in natural communities is difficult to quantify, mainly due to methodological limitations. In the past, nutrient assimilation in mixed plankton communities was best discriminated after water pre-filtration, i.e., size-fractionation. However, size-fractionation poorly separates plankton taxa of similar size or closely associated cells, often causes cell disruption and concurrent ammonium release, and destructs interactions between trophic levels [10, 11]. More recently, assimilation rates have been analysed by either stable-isotope probing [12] or flow cytometry combined with stable-isotope analyses [13] but both approaches are limited to most abundant taxa and cannot reveal single-cell activities. These methodological limitations can be resolved by secondary-ion mass spectrometry (SIMS) which enables single-cell analyses in mixed field populations after stable-isotope incubations [14]. Nutrient assimilation can thereby be differentiated between individual taxa and even cells while natural microbial interactions and nutrient concentrations remain relatively undisturbed.

Our study was motivated by two uncertainties in aquatic geomicrobiology. First, single-cell ammonium assimilation rates in natural marine plankton communities are poorly explored but crucial to elucidate taxa-specific nutrient preferences, assimilation rates and quantitatively important taxa for ammonium cycling. Second, previous studies have quantified the contribution of diazotrophs to primary production [e.g., ref. [15]] and N_2_-fixation as a new N-source, which becomes available as ammonium [7–9] or DON [9, 16–18]. Further, the transfer of new N from N_2_-fixing microbes to other phytoplankton, bacteria and zooplankton has been studied intensively during recent years in different environments, e.g., in the tropical South Pacific Ocean [19, 20], Gulf of Mexico and Caribbean Sea [17, 21], Southwest Pacific [22] and Baltic Sea [23–26]. However, the quantitative importance of new ammonium from N_2_-fixation in direct comparison to regenerated ammonium in field communities remains poorly known [but see ref. [27]]. In order to resolve these uncertainties, we studied ammonium cycling in N-depleted surface waters in the Baltic Sea, a semi-enclosed sea which has been monitored for more than 30 years [28]. Single-cell and large-scale observations have suggested that the new N-source from N_2_-fixation can be equal to or even exceed net N_2_-fixation [7, 8, 23, 29, 30]. Moreover, N-losses from the photic zone appear to be low and new N from N_2_-fixation is effectively transferred into pelagic food webs, explaining the observed increase in the total N inventory during summer [29, 31]. In the present study, we quantified ammonium processes, as well as N_2_-fixation and C-fixation in the photic zone using isotopic tracer incubations, mass spectrometry, ammonium analyses and microscopy, and linked our findings on the small-scale to existing meso-scale observations. The data collected foster our quantitative and mechanistic understanding of interlinked plankton growth and N-dynamics in marine waters, in which N-depletion, ammonium-based production and N_2_-fixation are prevalent.

## Materials and methods

### Study area and water sampling

Surface water (1–3 m) was collected with a water sampler (NM Tech AB, Stockholm, Sweden) at a coastal station in the Baltic Sea (station B1 of the Swedish National Marine Monitoring Program, N 58° 48’ 18 E 17° 37’ 52, depth 40 m) in June 2012 and August 2013. Sub-samples were 0.45 μm-filtered and stored at −20 °C for later nutrient analyses on a segmented flow nutrient analyser (ALPKEM O.I. Analytical Flow Solution IV, methods: phosphate #319528, nitrite + nitrate #319527, and nitrite #319527; with reporting limits of 16, 21 and 14 nmol L^−1^, respectively). Ammonium was analysed immediately (see below). Depth profiles of temperature, salinity, oxygen and light were recorded with a CTD (CTM577, Sea & Sun).

### Water incubations

Water was filled headspace-free into 1 L Duran® bottles. Three bottles were amended with ^15^N-ammonium (^15^NH_4_Cl, 98 atom% ^15^N, #299251 Aldrich) and ^13^C-DIC (^13^C-sodium bicarbonate, 98 atom%, #372382 Aldrich), another three bottles with ^15^N_2_ (98 atom% ^15^N, #364584 Aldrich) and ^13^C-DIC, and one bottle served as control without isotope additions. ^15^N-ammonium concentrations were 20–30 nM, equal to final ^15^N-label percentages of 5–46%. ^13^C-DIC was added to a final label percentage of 5% (methods described below). ^15^N-labelled N_2_ gas was added as pre-dissolved ^15^N_2_ [32], yielding final ^15^N-label percentages of 1% in 2012 and 9% in 2013. False N_2_-fixation rates due to ^15^N-contaminations in the gas bottles [33] could be excluded since the ^15^N_2_-amended water was tested negative for ^15^N-ammonium (analyses described below).

Water was sampled freshly 1 h before each incubation which took place at 0.5 m water depth in a mesocosm basin at in situ temperature and ambient light (Fig. S1) for approximately 3 h at four different times of the day (Table 1). The following sub-samples were taken at two (t0 and t3) or three time-points (t0, t1, t3) from the ^15^N_2_-ammonium and ^15^N-ammonium amended bottles, respectively: (i) ^*15*^*N-ammonium concentrations*, *and* ^*15*^*N*_*2*_*-labelling and* ^*13*^*C-labelling*–Triplicate sub-samples were preserved in 12 mL Exetainer® vials with 100 µL saturated ZnCl_2_ solution. (ii) *Bulk ammonium concentrations*–40 mL were transferred into acid-washed Falcon tubes plus 10 mL ortho-phthalaldehyde solution. Ammonium concentrations were determined on a fluorometer (Turner design, TD-700) after 6 h [34]. A 5-point calibration covering the expected concentration range (0–500 nM) was prepared, yielding a strong linear correlation between raw fluorescence and ammonium concentrations (*R*^2^ > 0.99). (iii) *Phytoplankton composition*–50 mL were preserved with Lugol solution for later microscopy. (iv) *Single-cell ammonium assimilation and C-fixation*–50 mL were preserved with paraformaldehyde (2% final concentration) and filtered onto polycarbonate membrane filters (0.22 µm GTTP, 25 mm, Merck Millipore) for (nano)SIMS analyses. (v) *Bulk N*_*2*_*-/C-fixation and ammonium assimilation*–500–600 mL were filtered onto pre-combusted GF/F filters (25 mm, Whatman) and analysed on an isotope-ratio mass spectrometer interfaced to an elemental analyser (EA-IRMS).

**Table 1:** Rates of ammonium cycling processes, N_2_-fixation and C-fixation

Date	Incubation period	Bulk NH_4_^+^ concentration (nM)	Gross NH_4_^+^ consumption (nmol N h^−1^ L^−1^)	Gross NH_4_^+^ production (nmol N h^−1^ L^−1^)	Net NH_4_^+^ rate (nmol N h^−1^ L^−1^)	NH_4_^+^ assimilation (nmol N h^−1^ L^−1^)		NH_4_^+^ turnover (h)	N_2_-fixation (nmol N h^−1^ L^−1^)	C-fixation (nmol C h^−1^ L^−1^)
						Analysed on GF/F	Analysed by SIMS			
28/29-June-2012										
	07:30–10:30	111 ± 44 (*n* = 3)	79 ± 24 (*n* = 9)	79 ± 24 (*n* = 9)	0	78 ± 29 (*n* = 9)	50 ± 34	1.4	9 ± 1 (*n* = 3)	167 ± 55 (*n* = 3)
	12:00–15:00	97 ± 9 (*n* = 3)	65 ± 11 (*n* = 9)	65 ± 11 (*n* = 9)	0	64 ± 18 (*n* = 9)	n/a	1.5	14 ± 1 (*n* = 3)	69 ± 5 (*n* = 3)
	16:30–19:30	79 ± 9 (*n* = 3)	67 ± 6 (*n* = 9)	67 ± 6 (*n* = 9)	0	51 ± 5 (*n* = 9)	n/a	1.2	22 ± 2 (*n* = 3)	257 ± 19 (*n* = 3)
	22:00–01:00	78 ± 17 (*n* = 3)	84 ± 17 (*n* = 9)	84 ± 17 (*n* = 9)	0	58 ± 13 (*n* = 9)	n/a	0.9	11 ± 4 (*n* = 3)	29 ± 1 (*n* = 3)
	**Daily integral [nmol d** ^**−1**^ **L** ^**−1**^ **]**		**1833**	**1833**	**0**	**1476**		**1.2 [h]**	**316**	**2367**
20/21-Aug-2013										
	07:30–10:30	568 ± 18 (*n* = 3)	171 ± 13 (*n* = 9)	120 ± 49 (*n* = 9)	−51	28 ± 2 (*n* = 9)	n/a	3.3	0.4 ± 0.3 (*n* = 3)	597 ± 7 (*n* = 3)
	14:00–17:00	45 ± 9 (*n* = 3)	94 ± 38 (*n* = 9)	94 ± 38 (*n* = 9)	0	36 ± 21 (*n* = 9)	n/a	0.5	3.5 ± 0.2 (*n* = 3)	1352 ± 8 (*n* = 3)
	18:30–21:30	32 ± 4 (*n* = 3)	67 ± 24 (*n* = 9)	67 ± 24 (*n* = 9)	0	25 ± 12 (*n* = 9)	32 ± 22	0.5	2.0 ± 0.3 (*n* = 3)	287 ± 13 (*n* = 3)
	23:30–02:30	33 ± 10 (*n* = 3)	92 ± 50 (*n* = 9)	92 ± 50 (*n* = 9)	0	37 ± 25 (*n* = 9)	n/a	0.4	0.79 ± 0.03 (*n* = 3)	17 ± 1 (*n* = 3)
	**Daily integral [nmol d** ^**−1**^ **L** ^**−1**^ **]**		**2561**	**2306**	**−256**	**809**		**1.2 [h]**	**36**	**11873**
	AVERAGE	130 ± 179 (*n* = 8)	90 ± 35 (*n* = 8)	84 ± 19 (*n* = 8)		47 ± 18 (*n* = 8)		1.2 ± 1.0 (*n* = 8)		

Bulk ammonium concentrations indicate concentrations before ^15^N-ammonium additions (20–30 nM). Data are given as mean ± s.d.

*n/a* not analysed

### Phytoplankton composition and biomass

Lugol-preserved samples were transferred into Utermoehl sedimentation chambers (Hydrobios) to identify and count phytoplankton taxa under an inverted light microscope (NIKON Eclipse Ti-U, ×150–400 magnification). Heterotrophic bacteria (DAPI-stained) and unicellular picocyanobacteria (autofluorescent) were counted on GTTP filters under a fluorescence microscope (Zeiss Axio Imager, ×1000 magnification). Cell sizes were measured on ≥40 cells for each taxon to reach representative mean values. Cellular biovolumes and biomass were calculated as specified in supplementary Table S1.

### Stable-isotope analyses

The ^15^N-label% of dissolved N_2_ was analysed by membrane-inlet mass spectrometry (MIMS^;^ GAM200, IPI, Bremen, Germany, relative precision ± 1%). The ^13^C-label% of dissolved inorganic carbon (DIC) was determined by trace gas isotope-ratio mass spectrometry (UC Davis California, US, precision ± 0.1‰). ^15^N-ammonium concentrations were measured after chemical conversion to N_2_ with alkaline hypobromite [35]. Production of ^15^N-nitrate and ^15^N-nitrite in ^15^N-ammonium incubations (i.e., nitrification) was quantified after conversion of nitrate to nitrite with cadmium, and nitrite to N_2_ with sulfamic acid [36] in samples from August 2013. ^15^N-standards were used to determine conversion factors. The N_2_ isotope ratios were analysed by gas chromatographic isotope-ratio mass spectrometry (GC-IRMS, concentration precision ± 5% for ^15^N-standards of 0–100 nM) on a Thermo Delta V isotope-ratio mass spectrometer [37]. Air was used as a standard and controls samples (without amendments) to determine the natural ^15 ^N mol fraction in the respective N-pools. GF/F filters were freeze-dried, fumed over HCl, pelletized into tin cups and analysed by EA-IRMS (UC Davis, precision ± 0.2‰ for ^13 ^C and ± 0.3‰ for ^15 ^N). Vienna PeeDee Belemnite and air served as C and N standards, respectively. Rates of bulk N_2_-fixation, C-fixation and net ammonium assimilation were calculated as described in supplementary Text S1. To extrapolate to rates per day, the rates measured at four different times of the day (Table 1) were linearly time-integrated over 24 h. Besides ammonium assimilation (accounting for ^15^N-PON on GF/F filters), we also calculated gross consumption (accounting for the actual ^15^N-ammonium decrease in the water) and production rates (Text S1). Ammonium production was specified to derive either from ammonium regeneration or from new ammonium released during N_2_-fixation. The latter was assumed to account for half of the N_2_-fixation rates, as shown for cells sampled concurrently with the ones herein [38] and during previous years [7, 23].

Due to ^15^N-ammonium additions, bulk concentrations increased by 5–46%, potentially stimulating ammonium assimilation. We therefore corrected all rates by accounting for ammonium uptake kinetics, as done previously [27, 39]. A half-saturation constant of 50 nM was assumed, in the upper range of 15–60 nM measured for natural plankton communities under N-depletion [27, 40, 41]. All equations and the resulting overestimations are given in supplementary Text S1.

### Secondary-ion mass spectrometry (SIMS and nanoSIMS)

^15^N-ammonium and ^13 ^C incorporation (after ^15^N-ammonium and ^13^C-DIC incubations) into single cells were analysed using two types of SIMS instruments (Cameca, France): IMS 1280 and NanoSIMS 50 L (at the Natural History Museum Stockholm and the MPI for Marine Microbiology, respectively). The NanoSIMS 50 L instrument offers a higher spatial resolution (50–100 nm) than the IMS 1280 (1000 nm) but the latter allows for a higher sample throughput and its higher primary-ion beam current facilitates the removal of consolidated cell walls. Accordingly, we analysed heterotrophic bacteria and unicellular picocyanobacteria (cf. *Synechococcus* spp.) exclusively on the NanoSIMS 50 L, and *Chaetoceros* sp. and dinoflagellates (*Dinophysis* sp., *Heterocapsa* sp.) on the IMS 1280. *Aphanizomenon* sp., *Dolichospermum* spp., *Nodularia spumigena*, colony-forming picocyanobacteria (*Aphanocapsa* sp., *Cyanodictyon* sp. and *Aphanothece paralleliformis*) and *Pseudanabaena* sp. were analysed with both instruments. Heterotrophic bacteria and *Synechococcus* were distinguished as free-living and attached (to other phytoplankton cells), as validated under a fluorescence microscope prior nanoSIMS analyses. Analyses were done on cells incubated during 07:30–10:30 in June 2012 and 18:30–21:30 in August 2013, since samples from those periods offered the highest cell abundances of the targeted plankton groups. SIMS analyses were conducted as presented elsewhere [38], except that diatoms and dinoflagellates were pre-sputtered with a higher Cs^+^ beam (4–6 nA for 240–480 s instead of 3 nA for 100 s) and imaged with 70 pA (instead of 40–60 pA) to remove the solid frustules/theca and penetrate into their rather thick cells. Regions of interest (ROIs) were drawn manually on the ^12^C^14^N ion images using the software Look@nanoSIMS [42] and WinImage (for IMS 1280 analyses). Isotope ratios for each ROI were averaged over 40–60 planes (nanoSIMS 50 L) and 100 planes (IMS 1280), and discarded if the standard error was >5%. Cells from control bottles without isotope additions served as standards. The ^15^N-atom% excess for control cells was on average 0.001 ± 0.001 (*n* = 235) and 0.000 ± 0.001 (*n* = 51), and the ^13^C-atom% excess 0.001 ± 0.001 (*n* = 235) and 0.000 ± 0.004 (*n* = 51) for analyses on the IMS 1280 and nanoSIMS 50 L, respectively. We mostly analysed >50 cells to reach representative mean values for each taxon [43, 44] (exceptions can be read from the number of replicates in Table 2).

**Table 2:** Single-cell ammonium assimilation and C-fixation rates of different plankton groups

	N-specific NH_4_^+^-assimilation		Cell-specific NH_4_^+^-assimilation		C-specific C-fixation		Cell-specific C-fixation		n	
	h^−1^		fmol NH_4_^+^ cell^−1^ h^−1^		h^−1^		fmol C cell^−1^ h^−1^			
	June 2012	Aug 2013	June 2012	Aug 2013	June 2012	Aug 2013	June 2012	Aug 2013	June 2012	Aug 2013
N_2_-fixing cyanobacteria										
*Aphanizomenon* sp.	0.003 ± 0.001	0.001 ± 0.001	1.1 ± 0.5	0.5 ± 0.2	0.012 ± 0.006	0.005 ± 0.002	25.7 ± 13.4	10.9 ± 5.2	65	53
	(0.001–0.007)	(0.0003–0.003)	(0.3–2.5)	(0.1–1.0)	(0.003–0.029)	(0.001–0.010)	(7.3–61.0)	(1.8–21.3)		
*Nodularia spumigena*	–	0.0008 ± 0.0004	n/p	0.6 ± 0.3	n/p	0.009 ± 0.005	n/p	46.5 ± 26.7	n/p	136
	–	(0.0001–0.003)		(0.1–2.2)		(0.002–0.021)		(9.5–107.3)		
*Dolichospermum* spp.	0.007 ± 0.003	0.003 ± 0.003	1.5 ± 0.9	0.6 ± 0.7	0.009 ± 0.002	0.011 ± 0.014	12.6 ± 5.8	14.7 ± 19.5	102	164
	(0.003–0.021)	(0.0001–0.014)	(0.6–4.5)	(0.02–3.1)	(0.005–0.016)	(0.001 ± 0.058)	(6.6–22.0)	(0.8–78.9)		
Non-N_2_-fixing cyanobacteria										
Filamentous *Pseudanabaena* sp.	0.029 ± 0.010	0.006 ± 0.002	0.8 ± 0.3	0.18 ± 0.06	0.005 ± 0.003	0.009 ± 0.005	1.0 ± 0.5	1.6 ± 1.0	27	69
	(0.015–0.051)	(0.003–0.012)	(0.4–1.5)	(0.08–0.35)	(0.002–0.017)	(0.001–0.026)	(0.4–3.2)	(0.2–5.0)		
Colonial picocyanobacteria *Aphanocapsa* sp./*Cyanodictyon* sp.	0.023 ± 0.010	0.008 ± 0.004	0.21 ± 0.11/0.12 ± 0.06	0.07 ± 0.04/0.04 ± 0.02	0.006 ± 0.003	0.008 ± 0.005	0.33 ± 0.19/0.19 ± 0.11	0.46 ± 0.34/0.27 ± 0.20	174	116
	(0.007–0.058)	(0.001–0.024)	(0.06–0.51)/ (0.04–0.30)	(0.01–0.22)/(0.01–0.13)	(0.001–0.012)	(0.002–0.024)	(0.03–0.69)/(0.02–0.40)	(0.12–1.40)/(0.07–0.81)		
Colonial picocyanobacteria *Aphanothece paralleliformis*	0.017 ± 0.005	0.005 ± 0.002	0.11 ± 0.04	0.03 ± 0.02	0.003 ± 0.001	0.005 ± 0.003	0.11 ± 0.06	0.21 ± 0.14	79	127
	(0.010–0.027)	(0.002–0.018)	(0.06–0.16)	(0.01–0.11)	(0.001–0.005)	(0.001–0.013)	(0.02–0.21)	(0.03–0.54)		
Unicellular picocyanobacteria cf. *Synechococcus* spp. (attached)	0.054 ± 0.023	0.015 ± 0.003	0.14 ± 0.07	0.04 ± 0.01	0.007 ± 0.001	0.025 ± 0.013	0.11 ± 0.03	0.42 ± 0.24	20	19
	(0.026–0.101)	(0.010–0.020)	(0.07–0.26)	(0.03–0.05)	(0.004–0.008)	(0.002–0.043)	(0.07–0.15)	(0.04–0.74)		
Unicellular picocyanobacteria cf. *Synechococcus* spp. (free-living)	0.044 ± 0.026	0.012 ± 0.005	0.11 ± 0.07	0.03 ± 0.02	0.008 ± 0.007	0.018 ± 0.009	0.13 ± 0.13	0.32 ± 0.16	71	126
	(0.008–0.101)	(0.002–0.029)	(0.02–0.26)	(0.01–0.07)	(0.001–0.033)	(0.001–0.047)	(0.01–0.57)	(0.02–0.82)		
Heterotrophic bacteria										
Heterotrophic bacteria (attached)	0.016 ± 0.010	0.022 ± 0.014	0.007 ± 0.004	0.009 ± 0.006	–	–	–	–	24	54
	(0.003–0.050)	(0.003–0.061)	(0.001–0.021)	(0.001–0.025)						
Heterotrophic bacteria (free-living)	0.011 ± 0.010	0.005 ± 0.006	0.005 ± 0.004	0.002 ± 0.002	–	–	–	–	302	86
	(0.002–0.057)	(0.0003–0.031)	(0.001–0.024)	(0.0001–0.013)						
Eukaryotes										
Diatom *Chaetoceros* sp.	0.034 ± 0.016	0.007 ± 0.002	13.9 ± 8.1	2.9 ± 1.2	0.020 ± 0.008	0.024 ± 0.011	55.9 ± 28.1	66.9 ± 38.1	65	23
	(0.005–0.081)	(0.003–0.011)	(2.0–33.6)	(1.0–4.6)	(0.002–0.036)	(0.007–0.047)	(6.4–97.5)	(18.6–128.8)		
Dinoflagellates (*Dinophysis, Heterocapsa*)	0.006 ± 0.005	n/a	n/a	n/a	n/a	n/a	n/a	n/a	12 (6+6)	n/a
	(0.001–0.017)									

Rates were measured for cells incubated during 07:30–10:30 in June 2012 and 18:30–21:30 in August 2013. Data are given as mean ± s.d. with their ranges in parentheses, n indicates the number of analysed cells

n/p cells not present, n/a not analysed

Activities measured by SIMS are expressed as element-specific assimilation rates (h^−1^), calculated as described in the supplementary (Text S1). Statistical differences between taxa were calculated with the post-hoc Tukey’s honest significant difference (HSD) test in R 3.3.0. To obtain cell-specific rates (fmol cell h^−1^), N-specific ammonium assimilation and C-specific C-fixation rates (h^−1^) were multiplied by cellular N-contents and C-contents (fmol cell^−1^), respectively. The C-contents and N-contents derived from empirical biovolume to biomass relationships (Table S1) which are routinely used for the long-term monitoring of Baltic Sea plankton [45] or have been measured directly for cyanobacteria at the sampling station [43]. Cell abundances were multiplied with cell-specific assimilation rates to quantify taxa-specific contributions to total ammonium assimilation. Uncertainties ( ± s.d.) in single-cell activities and taxa-specific contributions to total assimilation derived from combined uncertainties of each variable, following the laws of error propagation. To verify whether ammonium assimilation was diffusion-limited, we calculated maximum ammonium fluxes explained by mass transfer theory, i.e., diffusion-limited ammonium supply to single cells. Fluxes at *Synechococcus* cells were calculated from the analytical solutions of diffusion to a sphere [46] and at *Chaetoceros* for cylindrical cell-chains [47] (Text S1).

## Results

### Environmental data

Water temperature was 14.5 and 17.0 °C during sampling in June 2012 and August 2013, respectively; salinity was 6.2 and the mixed layer depth 25 m during both occasions (Fig. S2a). Nutrient concentrations were 0.03–0.57 µmol L^−1^ for ammonium (Tables 1), 0.02–0.04 µmol L^−1^ for nitrate + nitrite and 0.07–0.18 µmol L^−1^ for phosphate, similar to those reported by the Monitoring Program (Fig. S2b). POC and PON contents were 419 ± 60 µg C L^−1^ and 60 ± 8 µg N L^−1^ (*n* = 61) during June, and 380 ± 38 µg C L^−1^ and 64 ± 4 µg N L^−1^ (*n* = 60) during August (Fig. S3).

The bacterioplankton and phytoplankton biomass (ca 250 µg C L^−1^ during both samplings) comprised mainly Cyanobacteria (45–56% of the C-biomass), heterotrophic bacteria (23–49%), and to a lesser extent Dinophyta (0.3–12%) and Bacillariophyceae (4%, Fig. S3). The cyanobacterial biomass consisted of two orders (Chroococcales, 43–94% and Oscillatoriales, <1%) which did not fix N_2_ [38] and one N_2_-fixing order (Nostocales, 6–57%). Thus, 3–31% of the bacterioplankton and phytoplankton biomass were diazotrophs. Chroococcales were dominated by unicellular picocyanobacteria (>90%) which were classified as *Synechococcus*-type cells (cf. *Synechococcus* spp.).

### Single-cell ammonium assimilation and C-fixation

Single-cell activities are presented as element-specific rates (h^−1^) which are independent of cell size and thus allow to directly compare activities among different cell types and sizes. For instance, N-specific ammonium assimilation rates of 0.005 h^−1^ imply that 0.5% of the cellular N-content was assimilated per hour. Note that the assimilation rates are only valid for the time of the day when incubations for SIMS analyses were conducted while different activities can be expected during other times of the day.

**Fig. 1:**
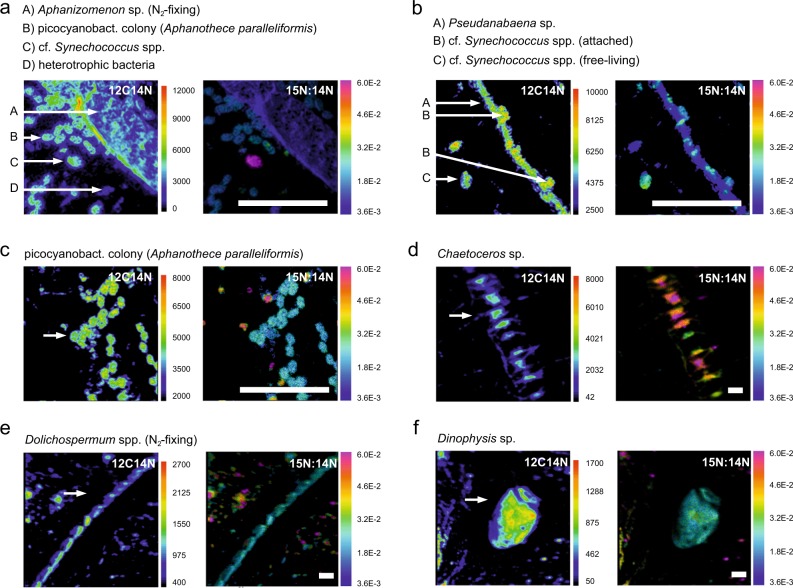
Secondary-ion mass spectrometer (SIMS) images of ^12^C^14^N counts (left panel) and ^15^N/^14^N isotope ratios (right panel) after ^15^N-ammonium incubations. Samples were analysed at high-resolution with the NanoSIMS 50 L (**a**–**c**) and at lower resolution with the IMS-1280 (**d**–**f**). Cell identification was done based on fluorescence microscope images taken prior SIMS analyses. White scale bars are 10 µm (note the different scale bars in panels **a**–**c** and **d**–**f**)

**Fig. 2:**
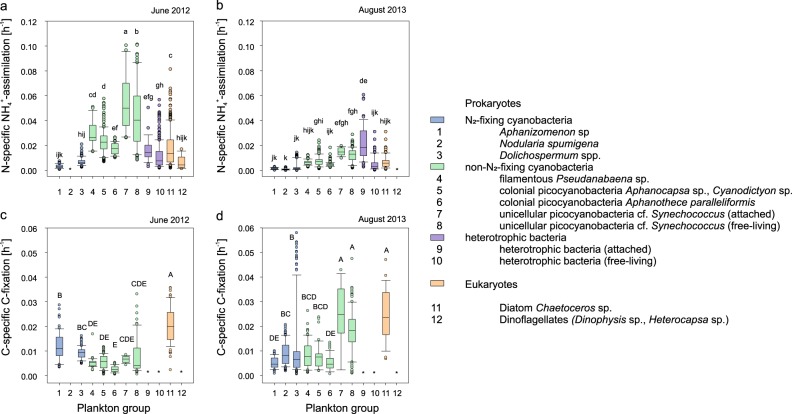
Single-cell ammonium assimilation (**a**, **b**) and carbon fixation (**c**, **d**) analysed by secondary-ion mass spectrometry. Rates were measured for cells incubated during 07:30–10:30 in June 2012 (**a**, **c**) and 18:30–21:30 in August 2013 (**b**, **d**). Significantly different rates are indicated by different letters (ammonium assimilation and carbon fixation rates were tested separately, shown by non-capitalised and capitalised letters, HSD-test, *p* < 0.05, Df = 1892 and 1279, respectively). Shown are the range (including 25 and 75% percentile, minimum, maximum and median) and outliers (circles). Note the different x-axes for ammonium assimilation and C-fixation. Asterisks indicate that no data are available. Details are listed in Table 2

**Fig. 3:**
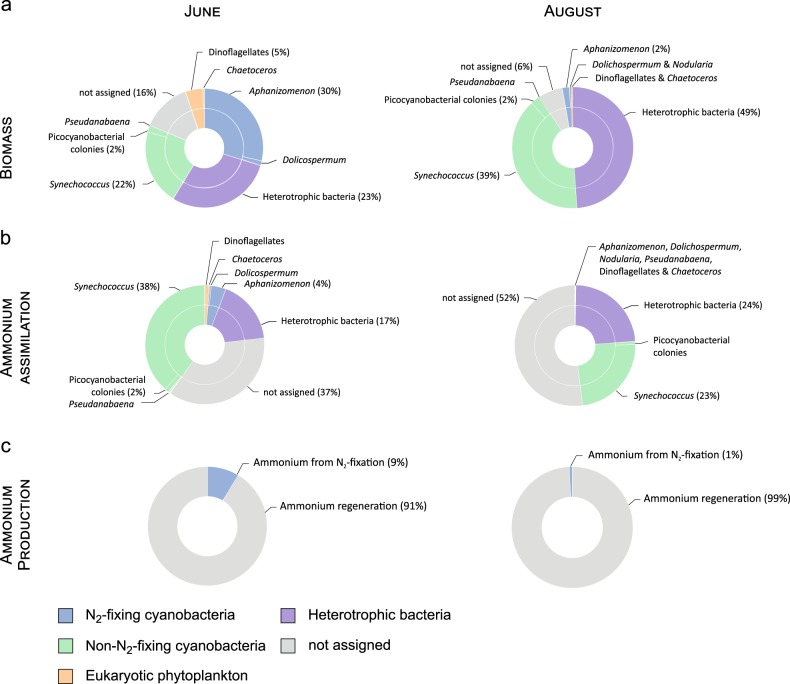
Relative carbon biomass (**a**) and ammonium assimilation (**b**) of bacterioplankton and phytoplankton in N-poor surface waters in the Baltic Sea. The not assigned biomass reflects the biomass of organisms which were microscopically identified and enumerated but not analysed by SIMS (see result section). Ammonium assimilation by the identified and analysed bacterio- and phytoplankton explained 48–63% of the total ammonium consumption (the remaining fraction is indicated as not assigned). Ammonium production resulted partly from N_2_-fixation but mostly from ammonium regeneration of unknown sources (**d**). Percentages are given in brackets (for contributions >1%). The relative standard deviation of the taxa-specific contributions in panels (**a**, **b**) was 59 ± 25% (*n* = 42). Dinoflagellates included *Dinophysis* and *Heterocapsa*

Taxa analysed with SIMS included N_2_-fixing cyanobacteria, non-N_2_-fixing cyanobacteria, heterotrophic bacteria and eukaryotes (Fig. 1), covering most of the C-biomass of the enumerated bacterio plankton and phytoplankton (≥84%). The taxa not analysed were less abundant (e.g., ciliate *Mesodinium*, diatom *Cyclotella*, Haptophyceae *Chrysochromulina*, and Cryptophyceae *Teleaulax* and *Plagioselmis*, Fig. S3). N-assimilation rates were highly variable, with mean N-specific assimilation rates ranging from 0.0008 to 0.054 h^−1^ (see Fig. 2 and Table 2 for details). Mean N-specific ammonium assimilation was lowest in filamentous N_2_-fixing cyanobacteria (0.0008–0.007 h^−1^) of which *Dolichospermum* had the highest rates, followed by *Aphanizomenon* and *Nodularia*. Cells of dinoflagellates (*Dinophysis*, *Heterocapsa*) were rare. Thus, their mean values obtained from only twelve cells (six per taxa) may poorly represent their entire population but indicated that ammonium assimilation was low (0.006 h^−1^). The quantitatively most significant groups for total assimilation were unicellular picocyanobacteria (*Synechococcus*) and heterotrophic bacteria—both small cells with high population biomass (Fig. 3) and high ammonium assimilation rates (mean values: 0.012–0.054 and 0.005–0.022 h^−1^, respectively, Table 2). *Synechococcus* accounted for 38 ± 31 and 23 ± 17%, and heterotrophic bacteria for 17 ± 18 and 24 ± 27% of the total assimilation in June 2012 and August 2013, respectively (Fig. 3c). Chain-forming diatoms (*Chaetoceros*) showed mean N-assimilation rates as high as 0.034 h^−1^ (Table 2). By comparison, theoretical ammonium assimilation rates constrained by diffusion-limited ammonium supply were 0.033–0.066 h^−1^ for chain-forming *Chaetoceros* (with 2–17 cells per chain) and 1.414 h^−1^ for unicellular *Synechococcus* (at ambient ammonium concentrations of 111 nM, as measured during the morning sampling in June 2012), indicating diffusion-limited assimilation in large *Chaetoceros* but no diffusion-limitation for *Synechococcus*.

Mean C-specific C-fixation rates ranged from 0.003 to 0.025 h^−1^ for phototrophic taxa. They were highest for *Chaetoceros* (0.020 h^−1^ in June 2012 and 0.024 h^−1^ in August 2013) and unicellular picocyanobacteria (0.025 and 0.018 h^−1^ for attached and free-living *Synechococcus*, respectively in August 2012). The remaining phototrophic cells showed lower C-fixation (mean: 0.003–0.012 h^−1^, Fig. 2 and Table 2).

### Community N_2_-fixation and C-fixation, and ammonium cycling

**Fig. 4:**
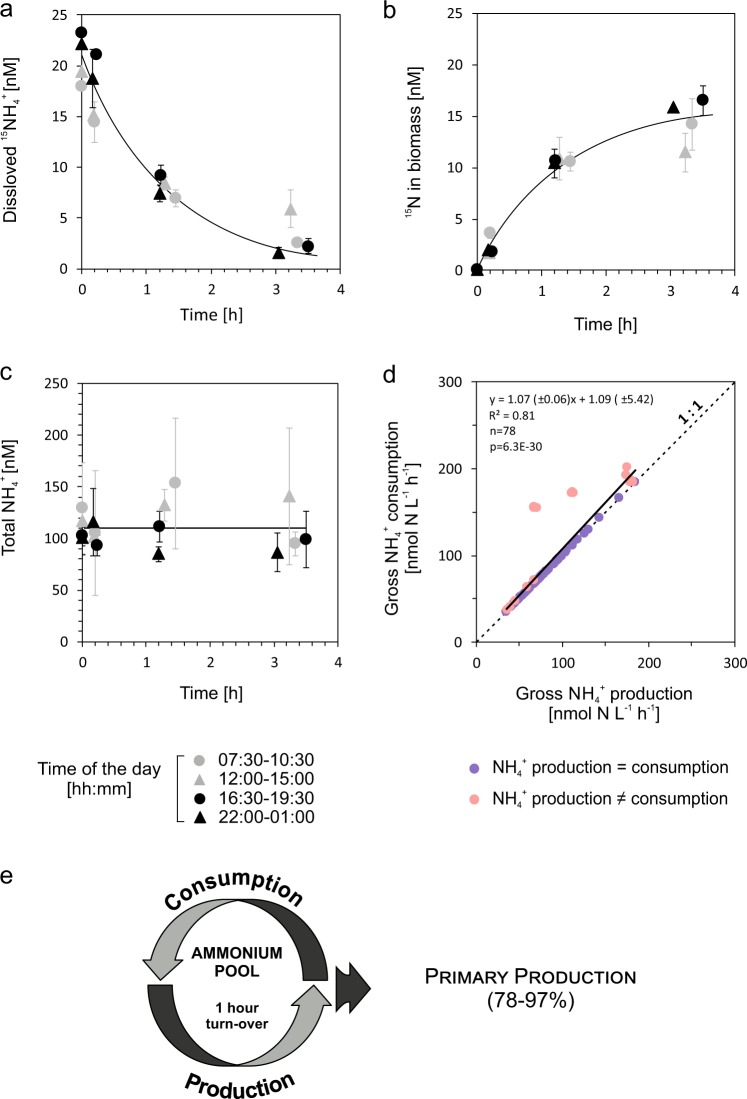
Ammonium dynamics in surface waters during N-depletion. Added ^15^N-ammonium decreased exponentially with time (**a**) and was mostly recovered in the biomass filtered onto GF/F filters (**b**), while total ammonium concentrations (measured after ^15^N-ammonium was added) remained at steady-state (**c**). Data are given as mean ± s.d. (*n* = 3) and are shown for incubations from June 2012 (**a**–**c**). Gross ammonium production and consumption rates (measured in June 2012 and August 2013) were positively correlated, following a close to 1:1 ratio (dashed line) (**d**). The rapid ammonium turn-over due to ammonium regeneration within 1 h could substantially sustain the N-demand for primary production (**e**)

N_2_-fixation rates were 0.4–21.9 nmol N h^−1^ L^−1^ (Table 1) with higher rates in June 2012 compared to August 2013 when the biomass of N_2_-fixing cyanobacteria was low (<10 µg C L^−1^, Fig. S3). New ammonium from daily-integrated N_2_-fixation potentially accounted for 9 and 1% of total ammonium production in June 2012 and August 2013, respectively, while the remaining ≥91% derived from regeneration. Added ^15^N-ammonium concentrations decreased exponentially over time. On average, 57 ± 28% (*n* = 16) of the consumed ^15 ^N was recovered as ^15^N-PON on GF/F filters. Bulk concentrations, however, remained at steady-state since gross consumption and production rates balanced each other (Fig. 4a–d). The turnover time through consumption was 1.2 ± 1.0 h. A diel pattern in ammonium processes was not evident (Table 1). Nitrification was not detectable since changes in ^15^N-nitrate/nitrite concentrations overtime were not significant (linear regression analyses, *p* > 0.10).

C-fixation rates were 17–1352 nmol C h^−1^ L^−1^, peaking during day-time and decreasing towards midnight (Table 1). In August 2013, 97% of the N-demand for diurnal C-fixation was supported by ammonium regeneration (Fig. 4e) and 2% by N_2_-fixation (assuming Redfield ratio and given that 24% of the produced ammonium was assimilated by heterotrophic bacteria and not by primary producers, as shown in Fig. 3b). In June 2012, ammonium production even exceeded the N-demand but C-fixation was probably photo-inhibited (at up to 1250 µmol photons s^−1^ m^−2^, Fig. S1) since C-fixation rates of the same plankton community measured in a parallel study at lower light were five times as high as those measured herein [38]. Assuming that actual C-fixation was five times higher, the N-demand for diurnal C-fixation would have been sustained to 78% by ammonium regeneration (Fig. 4e) and to 16% by N_2_-fixation (at Redfield ratio and given that 17% of the ammonium were assimilated by heterotrophic bacteria).

## Discussion

### Tight ammonium coupling and N_2_-fixation sustain long-term N-availability for primary production

Primary production based on N_2_-fixation and ammonium regeneration often dominates across diverse aquatic environments [1–3]. At the herein sampled coastal area, ammonium production derived mostly from regeneration and less from recent N_2_-fixation (Fig. 3c). Yet, parts of the regenerated ammonium may have its origin in N_2_-fixation hours, days or weeks prior to our sampling. Additional N may have been supplied as DON released from diazotrophs [9, 16, 18, 48]. Recently, amino acids have been shown to be newly synthetised during N_2_-fixation, and incorporated into bulk PON at rates of 0.5–7.0 nmol L^−1^ h^−1^ during summer in the Baltic Sea [18]. Those rates correspond to 1–8% of the ammonium consumption rates measured in our study (on average 90 nmol L^−1^ h^−1^, Table 1). Ammonium regeneration was the predominant N-source for the autotrophic plankton community (78–97%), as shown earlier in the Baltic Sea [49]. By comparison, new production, i.e., N_2_-fixation, supported 2–16% of the N-demand for primary production, in rough agreement with our previous estimate that N_2_-fixation supports 21% of the C-fixation over summer in the euphotic zone of the Northern Baltic Proper [38].

Interestingly, primary production rates were as high as those typically measured during spring (Swedish Monitoring Program) when new production is based on nitrate. High primary production rates despite low nutrient concentrations were reconciled by a tight coupling of production and consumption rates, following a close to 1:1 ratio (Fig. 4d) [see also refs. [3, 50]]. Therefore, the *de facto* N-availability was extended by fast turnover times (on average 1 h), which are common in N-depleted marine estuaries and lakes [51–53] but shorter than under N-rich conditions [51, 54]. The high N-retention through regeneration and re-assimilation in the photic zone combined with low sedimentation losses, e.g., of slow-sinking picocyanobacteria and buoyant N_2_-fixing cyanobacteria [55] supports an increase of the total N inventory due to new N from N_2_-fixation [29, 31]. Thus, diazotrophic-derived and regenerated N is effectively retained and accumulated in the upper mixed layer from early towards late summer when the food demand by fish is highest [24].

### Quantitative ammonium assimilation assays: SIMS vs. EA-IRMS

Ammonium consumption and production rates were 65–171 nmol N L^−1^ h^−1^ (Table 1), similar to those reported for coastal areas but higher than those previously measured in the Baltic Sea [56] and in worldwide oceanic and estuarine systems [summarised in refs. [4, 6]]. As a novelty—compared to numerous black-box-experiments, dating as far back as half a century ago [57], and also more recent SIMS-based ammonium analyses in freshwater systems [58], marine sediments [59] and coral–dinoflagellates symbioses [60]—we quantified ammonium assimilation for major taxa of the bacterioplankton and phytoplankton in marine waters. Recently, single-cell analyses by SIMS could fully explain community N_2_-fixation [38] measured on GF/F filters by EA-IRMS when large phytoplankton dominated the activity. In the herein presented study, taxa analysed by SIMS explained 48–63% of the gross ammonium consumption, whereas assimilation by cells collected onto GF/F filters explained 37–98%. Hence, both approaches (EA-IRMS and SIMS) did not fully explain total ammonium consumption rates. Small heterotrophic bacteria greatly contributed to community biomass and ammonium assimilation (Fig. 3a, b) but GF/F filter have been shown to poorly retain bacterioplankton [56], thus underestimating their activities [27, 61]. Our SIMS data may underrate single-cell assimilation rates of the picoplankton due to uncertainties in their cellular N-contents and uptake kinetics. To correct assimilation rates for any potential stimulation after ^15^N-ammonium additions, we used a half-saturation constant value of 50 nM, which might be lower for small heterotrophic bacteria and picocyanobacteria, and potentially underestimate our rates after correction. Bulk C-fixation was indeed not stimulated by ^15^N-ammonium additions, as implied from similar C-fixation rates measured after ^15^N_2_ and ^15^N-ammonium incubations (Fig. S4). Numerically inconspicuous taxa not analysed by SIMS might have also contributed to ammonium assimilation disproportionally to their population biomass, as shown for anaerobic bacteria [58] and diatoms [62]. The mismatch of ammonium assimilation and consumption might also be explained by nitrification but we could not detect any significant rates of this process. Nitrification was also not detectable in previous studies in N-depleted Baltic Sea surface waters during summer [63, 64] and nitrifiers are generally outcompeted by phytoplankton under nitrate-replete regimes [65]. Consistently, high nitrification rates have been measured recently in other coastal areas of the Baltic Sea when nitrate concentrations were substantially higher (>0.7 µmol L^−1^) than at our sampling station [66, 67].

### Single-cell ammonium assimilation by diazotrophs

Using SIMS, we could analyse in situ assimilation rates across various functional plankton taxa with different or even similar cell sizes. Intriguingly, filamentous N_2_-fixing cyanobacteria did not substantially take up ammonium which is supported by long-term observations of natural isotopic compositions of these cyanobacteria in the Baltic Sea [68]. *Aphanizomenon* contributed maximally 4 ± 3% to the total ammonium assimilation although they accounted for up to 30 ± 12% of the C-biomass (Fig. 3a, b). Ammonium assimilation rates were low (Table 2), as already shown for *Aphanizomenon* sp., presumably due to colony-formation which reinforces diffusion-limited ammonium transport towards cells [23]. In a parallel study to that in June 2012, N_2_-fixation rates were as fast as 0.023–0.097 h^−1^ for *Aphanizomenon* and *Dolichospermum* [38] while herein measured ammonium assimilation rates were 0.0008–0.007 h^−1^. Therefore, their potential cellular N-turnover was more than one order of magnitude faster by N_2_-fixation than by ammonium assimilation. The low ammonium assimilation by filamentous N_2_-fixing cyanobacteria is also supported by the observation that cyanobacterial colonies release significant amounts of ammonium [7, 8] and DON [16, 18], depending on their energy reserves. Colony-forming cyanobacteria such as the Baltic Sea strains and the widespread *Trichodesmium* may indeed re-assimilate only parts of their newly released N [69] while the remaining parts may benefit attached microbiota and co-occurring plankton [20, 23, 25, 70].

### Single-cell ammonium assimilation by non-diazotrophs

Single-cell ammonium and C-assimilation rates were highly variable, often differing by one order of magnitude among diverse taxa and even single species (Fig. 2). Such phenotypic heterogeneity in metabolism can result from (i) diffusion-limited nutrient assimilation in chain- or colony-forming species in which cells are exposed to distinct chemical microenvironments [44], (ii) variable substrate preferences of cells within the same population [71, 72] or (iii) metabolic versatility within cell populations to cope with substrate fluctuations [73].

Colony-forming picocyanobacteria and *Pseudanabaena* have been considered as potential N_2_-fixers [74, 75]. However, recent SIMS-based analyses did not confirm substantial N_2_-fixation with rates as low as 0.001–0.004 h^−1^ of those taxa in the Baltic Sea [38]. Instead, they seem to preferably assimilate ammonium at rates of 0.006–0.029 h^−1^ (Table 2). Total ammonium assimilation was dominated by autotrophic picocyanobacteria and heterotrophic bacteria (Fig. 3b) which apparently competed for the same N-source. Their assimilation rates agreed well with recent studies on a single-cell level for both taxa [23] and on a community level for prokaryotes [76] and specifically heterotrophic bacteria [77, 78]. Single-cell assimilation rates of *Synechococcus* were also similar to those reported from the Pacific Ocean [72] and to doubling times of ~1–2 days (equivalent to net N-assimilation rates of 0.021–0.042 h^−1^) measured for entire picocyanobacterial communities during summer in the Baltic [79, 80]. Such fast assimilation rates may substantially support higher trophic levels, since picocyanobacteria are actively grazed by zooplankton in the Baltic Sea [81, 82]. Heterotrophic bacteria usually regenerate ammonium through the degradation of dissolved organic matter (DOM), i.e., ammonification. Still, their ammonium assimilation rates were high, comparable to those of phototrophic, non-N_2_-fixing cells (Fig. 2 and Table 2). Potentially, some cells received their ^15^N-enrichment not directly from ^15^N-ammonium assimilation but rather from ^15^N-DON released after ^15^N-assimilation by the bacterioplankton or phytoplankton. DON greatly supports plankton nutrition [83, 84] and its release accounts for on average 20–30% of the ammonium assimilation [85, 86]. However, only parts of the recently released DON may be bioavailable [87] and DON turnover times are rather long, in the order of days [18, 88, 89]. We thus consider the ^15^N-enrichment in cells due to recently excreted ^15^N-DON as minor during our 3-h incubations. In the Baltic Sea, the C:N ratios of DOM are >10 [90] while bacterial C:N ratios are commonly 3.7 [91] with mean bacterial growth efficiencies (BGE) of 0.34 [92]. Such combination of high BGE, high substrate C:N ratio and low bacterial C:N ratio implies net N assimilation rather than release by heterotrophic bacteria [93]. Regenerated ammonium can also derive from, e.g., zooplankton grazing, release by phytoplankton, viral infections or cell lysis [6]. Teasing these processes apart is challenging but should be targeted in future studies, to untangle the herein reported large fraction of ammonium regeneration of unknown sources (Fig. 3c).

### Nutrient acquisition in small vs. large cells

Small cells are generally believed to grow faster than large cells at low steady-state nutrient concentrations because of their higher cell surface-to-volume ratios [94]. Nevertheless, we measured similar ammonium-assimilation and even C-assimilation rates (h^−1^) in small picocyanobacteria and large chain-forming diatoms (Table 2). *Chaetoceros* even showed N-assimilation rates similar to those predicted by theoretical diffusion-limited ammonium supply. N-assimilation rates of *Chaetoceros* based on ammonium during June (0.034 ± 0.016 h^−1^) were also similar to those based on nitrate (0.023 ± 0.015 h^−1^ at 0.3 µM) during diffusion-limited growth at the end of the spring bloom at the same sampling station [44]. Diatoms may thus compete well for dissolved inorganic N not only in upwelling, nitrate-rich areas but also in the N-poor regions. In fact, diatom diversity is comparable in oligotrophic and nutrient-rich areas with *Chaetoceros* as the most abundant and diverse genus [95], and diatoms have been shown to compete well for N released from N_2_-fixation [20, 23]. A recent study has also demonstrated that *Chaetoceros* contributed ≥20% to the total C and N assimilation under N-depleted conditions although it accounted for only 6% of the phytoplankton biomass [62]. However, high C-specific and N-specific assimilation rates of *Chaetoceros* contradicted their low population biomass (<0.2 µg C L^−1^) in this study, which remains enigmatic at present. Assimilation rates measured by SIMS are a relative measure of the elemental turn-over within cells, independent on cell sizes. Those rates may reflect single-cell growth rates, yet they may not necessarily correlate to actual biomass built-up. Rates obtained from SIMS analyses assume that the CN-biomass is evenly distributed in cells, which may not always hold true. For instance, nutrient-storing vacuoles can cover large parts of the cell volume in diatoms but account for proportionally little biomass—a structural feature which may overestimate N-growth rates of diatoms when using SIMS [96]. Moreover, the population size of *Chaetoceros* might have been limited by other nutrients than N and/or moderated by fast sinking as indicated by their proportionally high retrieval in sediment traps [97] and high grazing pressure from zooplankton [24].

In conclusion, our experimental conditions resembled growth conditions for plankton communities—including N-depletion, ammonium regeneration and N_2_-fixation—that currently predominate in marine waters and may even intensify in the future [98–100]. Under these conditions, eukaryotic diatoms showed a fast C-turnover and N-turnover on a single-cell level but minor population biomass. In contrast, prokaryotic picoplankton of different trophic levels, i.e., heterotrophic bacteria and autotrophic picocyanobacteria quickly turned over their cellular C-content and N-content by C-fixation and ammonium assimilation, respectively, and also dominated the community biomass, thereby facilitating rapid nutrient dynamics in N-depleted marine systems.

## Supplementary information

Supplementary
