# Supplementary material for: Untangling hidden nutrient dynamics: rapid ammonium cycling and single-cell ammonium assimilation in marine plankton communities
Source: ISME J. 2019 Mar 25;13(8):1960–74. doi: 10.1038/s41396-019-0386-z (PMC6776039; doi:10.1038/s41396-019-0386-z)
Supplement: Supplementary file 1 — Supplementary [file 41396_2019_386_MOESM1_ESM.pdf]

SUPPLEMENTARY INFORMATION

Untangling hidden nutrient dynamics: Rapid ammonium cycling and single-cell ammonium assimilation in marine plankton communities

Isabell Klawonn, Stefano Bonaglia, Martin J. Whitehouse, Sten Littmann, Daniela Tienken, Marcel M.M. Kuypers, Volker Brüchert, Helle Ploug

Text S1 Material and Methods

Calculations for bulk ammonium cycling, N_2- and C-fixation

Rates ρ of bulk N_2-fixation, C-fixation and net ammonium assimilation were calculated following reference [1], as

$$\rho_{N_2\text{-fixation}} = \frac{A^{PN}_{sample} - A^{PN}_{control}}{A_{N2} - A^{PN}_{control}} \times \frac{[PN]}{t} \quad (1)$$

$$\rho_{C\text{-fixation}} = \frac{A^{PC}_{sample} - A^{PC}_{control}}{A_{DIC} - A^{PC}_{control}} \times \frac{[PC]}{t} \quad (2)$$

$$\rho^{net}_{NH_4^+ \text{assimilation}} = \frac{A^{PN}_{sample} - A^{PN}_{control}}{R} \times \frac{[PN]}{t} \quad (3)$$

where *A* is the isotope atom% in the dissolved N_2 pool (A_{N2}), DIC pool (A_{DIC}) or particulate material (A_{PN}, A_{PC}); *PN* and *PC* are the amounts of particulate N and C, respectively, and *R* the ^{15}N-ammonium atom% excess in the dissolved ammonium pool. *R* was calculated by accounting for the exponential decrease in ^{15}N-ammonium concentrations due to concurrent ammonium assimilation and regeneration following references [2].

$$R = \frac{R_0}{kt}\,[1\text{-}\exp(\text{-}kt)] \qquad (4)$$

where R_0 is the ^{15}N-ammonium atom% excess at time zero, t the incubation time and k a constant determined from the exponential decrease of R over time:

$$R = R_0 \times \exp^{-kt} \qquad (5)$$

Besides ammonium assimilation rates (accounting for ^{15}N-PON on GF/F filters, equation 3), we also calculated gross consumption rates which accounted for the actual ^{15}N-ammonium decrease in the water

$$\rho^{\text{gross}}_{NH_4^+\text{consumption}} = \frac{(C_0 - C_t)}{(R \times t)} \qquad (6)$$

where C_t and C_0 are the ^{15}N-ammonium concentrations measured at time zero and time t. Production rates were calculated differently for incubations during which ammonium concentrations either remained stable (equation 7) or changed significantly over time (equation 8).

$$\rho^{\text{gross}}_{NH_4^+\text{production}} = k \times P \qquad (7)$$

$$\rho^{\text{gross}}_{NH_4^+\text{production}} = \rho^{\text{gross}}_{NH_4^+\text{consumption}} + \left(\frac{P_t - P_0}{t}\right) \qquad (8)$$

where P is the mean ammonium concentration (initial ^{14}N- plus added ^{15}N-ammonium) during incubations and $(P_t\text{-}P_0)$ the difference in ammonium concentrations between time points. Ammonium production was specified to derive either from ammonium regeneration or from new production as ammonium release during N_2-fixation. The latter was assumed to account for half of the N_2-fixation rates, as shown for cells sampled concurrently with the ones herein [3] and during previous years [4, 5]. Parts of the new N might have also been released as DON (see discussion in main text). Rates were calculated for the initial ≤2 h of each incubation since ^{15}N-ammonium concentrations decreased rapidly, often getting depleted before the final sampling point (especially

in August). The turnover time of ammonium was defined as $P_{0\text{-}15N}$ (initial ammonium concentration before ^{15}N-ammonium addition) divided by gross production rates.

Calculations of single-cell assimilation rates

Single-cell N-specific ammonium assimilation and C-specific C-fixation rates after SIMS analyses were calculated as

$$\text{N-specific } NH_4^+ \text{ assimilation } \left(h^{-1}\right) = \frac{A_{\text{sample cell}} - A_{\text{control cell}}}{R \times t} \qquad (9)$$

$$\text{C-specific C-fixation } \left(h^{-1}\right) = \frac{A_{\text{sample cell}} - A_{\text{control cell}}}{(A_{\text{DIC sample}} - A_{\text{DIC control}}) \times t} \qquad (10)$$

where A is the ^{15}N- and ^{13}C-atom%, respectively.

Note that single-cell rates may be closer to gross rather than net activities, due to our short incubation times and the coupling of assimilation and release processes.

Due to the ^{15}N-ammonium additions, bulk concentrations increased by 5–46%, potentially stimulating ammonium assimilation. We therefore corrected all rates by accounting for ammonium uptake kinetics, as done previously [6, 7]:

$$\rho^{\text{corrected}}_{NH_4^+ \text{ assimilation}} = \frac{\rho^{\text{measured}}_{NH_4^+ \text{assimilation}}}{P_0/(K_s+P_0)\times(K_s+P_{0\text{-}15N})/P_{0\text{-}15N}} \qquad (11)$$

where $P_{0\text{-}15N}$ and P_0 are the ambient ammonium concentrations before and after ^{15}N-ammonium additions, respectively, and Ks is the half-saturation constant which we assumed to be 50 nM, in the upper range of literature values of 15–60 nM [7-9]. The resulting overestimations were 8% for June incubations (after 20±5% ^{15}N-ammonium additions, n=36), 0.4% for the morning incubations

in August 2013 (5.0±0.2% ^{15}N-additions, n=9) and 39% for the remaining incubations in August (46±6% ^{15}N-additions, n=27). All ammonium consumption, production and assimilation rates (including bulk and single-cell data) were corrected according to those percent overestimations.

Ammonium fluxes constrained by diffusion-limited ammonium supply to single *Synechococcus* cells were calculated from the analytical solutions of diffusion to a sphere [10]

$$Q_{max} = 4\pi D r_0 (C_\infty - C_0) \qquad (12)$$

where Q_{max} is the potential ammonium uptake rate (nmol s^{-1}) of a cell with the equivalent spherical radius r_0, D the ammonium diffusion coefficient in water (1.57 x 10^{-5} cm^2 s^{-1} at 16°C and salinity of 6) [11], C_0 the ammonium concentration at the cell surface (assumed to be zero) and C_∞ the concentration in the ambient water. Maximum ammonium fluxes to *Chaetoceros* were calculated for cylindrical cell-chains [12]

$$Q_{max} = \left[8 + 6.95\left(\frac{l}{d}\right)^{0.76}\right] r_0 \times D \times (C_\infty - C_0) \qquad (13)$$

where l is the length and d the diameter of diatom chains with at least 2 and up to 17 cells (as observed under the microscope).

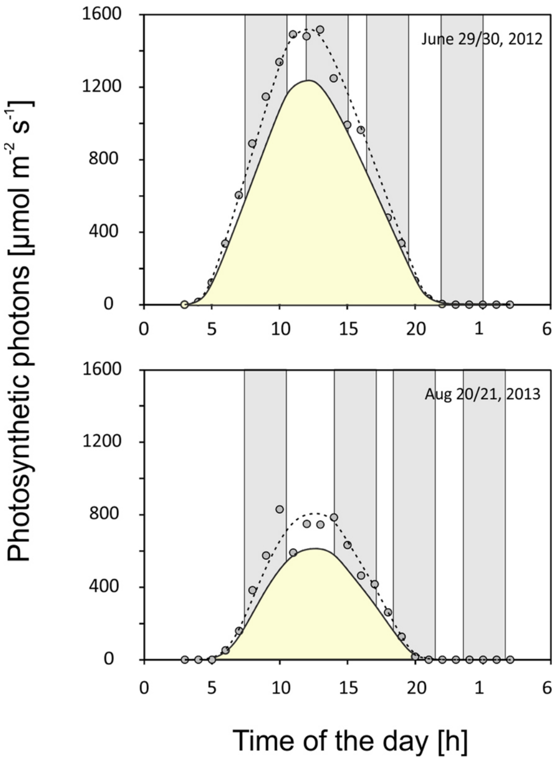

Figure S1. Solar irradiance during incubations in June 2012 and August 2013. Light intensities in air are shown as grey circles/dashed outline, while light at 0.5 m water depth is highlighted in yellow. A light attenuation coefficient of 0.37 was applied. Incubation periods are highlighted as grey-shaded bars. Data derive from the Swedish Meteorological and Hydrological Institute (SMHI) and were produced with support from the Swedish Radiation Protection Authority and the Swedish Environmental Agency.

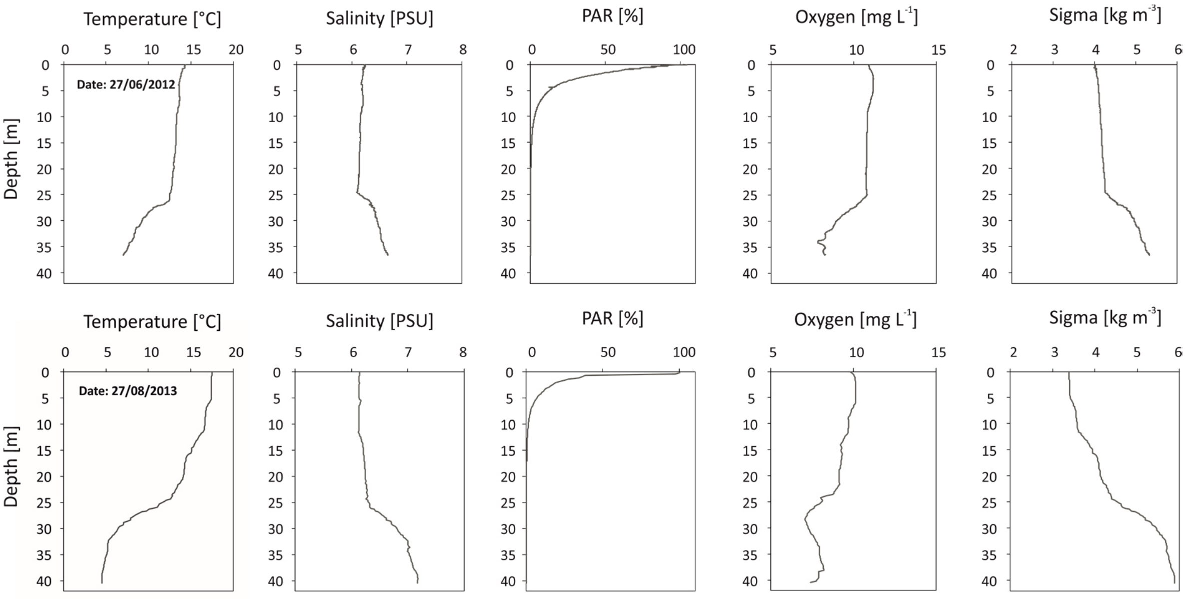

Figure S2a. CTD profiles recorded at station B1 on 27/06/2012 (upper panel) and 27/08/2013 (lower panel). PAR - Photosynthetically active radiation

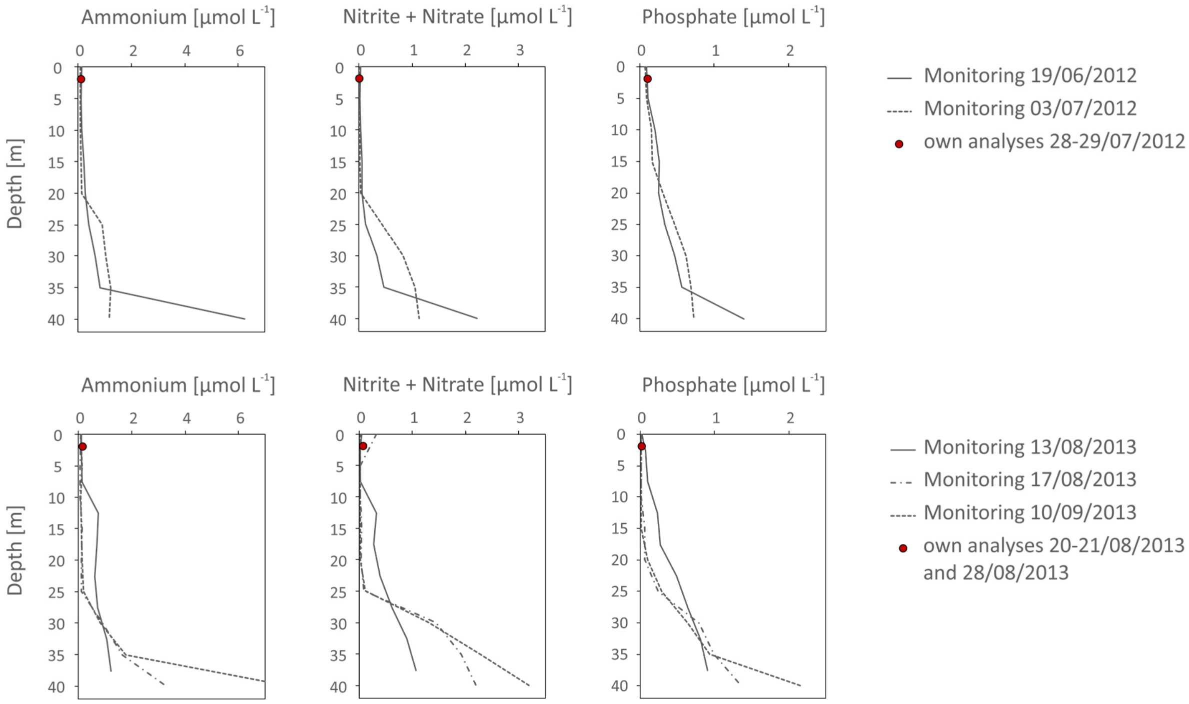

Figure S2b. Nutrient concentration profiles at Station B1, recorded by the Swedish National Marine Monitoring Program (SNMMP), at dates which coincided closest with our own water sampling. Nutrient data from the actual incubation water (sampled at 1–3 m depth, red data points) are plotted in comparison to the profiles (black lines). Monitoring data were extracted from the public database available online

(http://www.smhi.se/klimatdata/oceanografi/havsmiljodata/marina-miljoovervakningsdata).

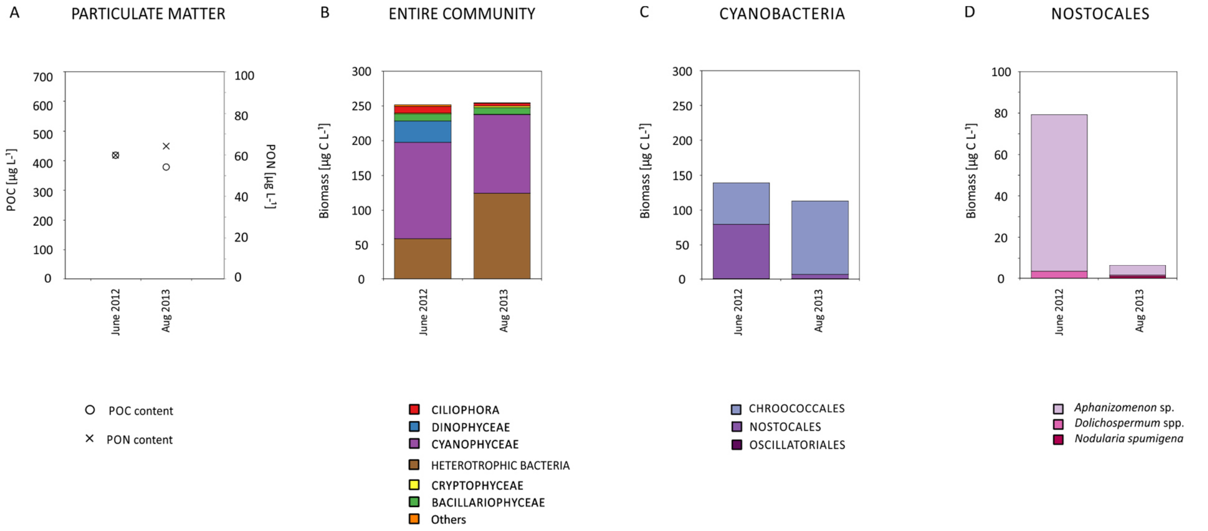

Figure S3. Particulate organic carbon and nitrogen (POC and PON measured on GF/F filters), as well as C-biomass and community composition of the bacterio- and phytoplankton during incubations. The bacterio- and phytoplankton biomass (ca 250 µg C L^{-1} during both samplings) comprised mainly Cyanobacteria (45–56% of the C-biomass), heterotrophic bacteria (23–49%) and to a lesser extent Dinophyta (0.3–12%) and Bacillariophyceae (4%), Ciliophora (1–4%, including only *Mesodinium rubrum* as autotrophic ciliate), Cryptophyceae (0.4–0.9%) and *others* (<1%, Haptophyceae, Zoomastigophora, Chlorophyta).

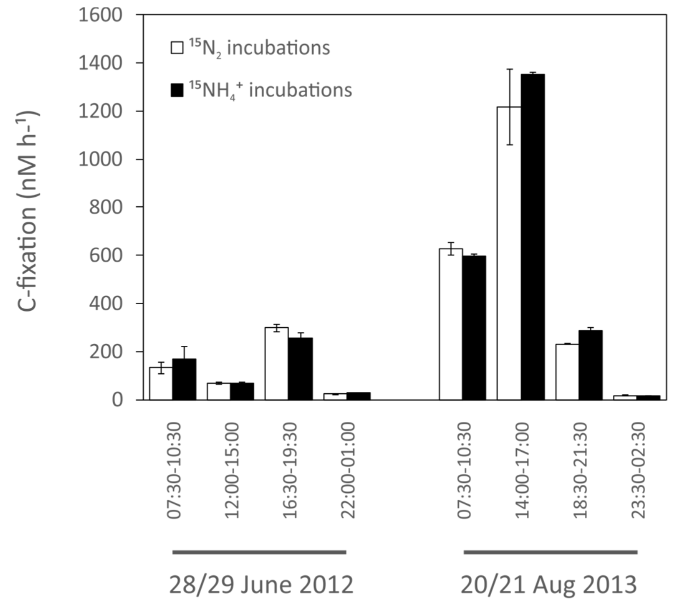

Figure S4. Carbon fixation rates measured during June 2012 and August 2013 in parallel incubated bottles enriched with $^{15}N_2$-gas or ^{15}N-ammonium. Similar rates in both incubations indicated no stimulation of C-fixation due to ammonium additions. Shown are average ± stdev (*n*=3).

Table S1. Dimensions and C-/N-contents of the analysed phyto- and bacterioplankton. The cellular biovolume and biomass of the various plankton groups were calculated *sensu* [13, 14] and the HELCOM guidelines used within the Baltic Sea monitoring program [15, 16]. ESD – Equivalent spherical diameter

	**Cell size (µm)**			**ESD (µm)**	**Biovolume (µm^{-3})**	**N-content (pmol N cell^{-1})**	**C-content (pmol C cell^{-1})**	**Reference Calculation of C and N content**
	**d1**	**h**	**d2**					
Aphanizomenon	4.3	8.4		6.2	122.0	0.34±0.07	2.13±0.41	[13]
N. spumigena	10.4	3.5		8.3	297.3	0.82±0.17	5.19±1.09	[13]
Dolichospermum	5.3			5.3	78.0	0.22±0.08	1.36±0.51	[13]
Pseudanabaena	1.5	6.9		2.9	12.2	0.029±0.002	0.19±0.02	[16]
Colonial picocyanobacteria *Aphanocapsa*	1.5			1.5	1.8	0.009±0.002	0.059±0.016	[14]
Colonial picocyanobacteria *Cyanodictyon*	1.0	1.2		1.2	0.9	0.005±0.001	0.034±0.010	[14]
Colonial picocyanobacteria *A. paralleliformis*	0.9	1.8		1.3	1.1	0.006±0.002	0.041±0.011	[14]
Unicellular picocyanobacteria *Synechococcus*	0.7	1.1		0.9	0.4	0.003±0.001	0.017±0.004	[14]
Heterotrophic bacteria	0.45	0.60		0.52	0.072	0.0004±0.0001	0.0017±0.0003	[16]
Diatom *Chaetoceros*	13.0	5.1	6.6	8.7	344	0.4±0.1	2.7±0.9	[16]
Dinoflagellates *Dinophysis*	34.0	48.0	24.3	34.1	20753	30.0±4.8	198.9±32.1	[16]
Dinoflagellates *Heterocapsa*	16.5	23.1		14.7	1646	2.8±1.7	18.9±11.0	[16]

References

1. Montoya JP, Voss M, Kähler P, & Capone DG (1996) A simple, high-precision, high-sensitivity tracer assay for N_2 fixation. *Appl. Environ. Microbiol.* 62(3):986-993.

2. Glibert PM, Lipschultz F, McCarthy JJ, & Altabet MA (1982) Isotope dilution models of uptake and remineralization of ammonium by marine plankton *Limnol. Oceanogr.* 27(2)(2):639-650.

3. Klawonn I, Nahar N, Walve J, Andersson B, Olofsson M, Svedén JB *et al.* (2016) Cell-specific nitrogen- and carbon-fixation of cyanobacteria in a temperate marine system (Baltic Sea). *Environ. Microbiol.* 18(12):4596-4609.

4. Adam B, Klawonn I, Svedén JB, Bergkvist J, Nahar N, Walve J *et al.* (2016) N_2-fixation, ammonium release and N-transfer to the microbial and classical food web within a plankton community. *ISME J.* 10(2):450-459.

5. Ploug H, Adam B, Musat N, Kalvelage T, Lavik G, Wolf-Gladrow D *et al.* (2011) Carbon, nitrogen and O_2 fluxes associated with the cyanobacterium *Nodularia spumigena* in the Baltic Sea. *ISME J.* 5:1549–1558.

6. Rees AP, Joint I, & Donald KM (1999) Early spring bloom phytoplankton-nutrient dynamics at the Celtic Sea Shelf Edge. *Deep-Sea Res. Pt I* 46(3):483-510.

7. Raimbault P & Garcia N (2008) Evidence for efficient regenerated production and dinitrogen fixation in nitrogen-deficient waters of the South Pacific Ocean: impact on new and export production estimates. *Biogeosciences* 5(2):323-338.

8. Sahlsten E (1987) Nitrogenous nutrition in the euphotic zone of the Central North Pacific Gyre. *Mar. Biol.* 96(3):433-439.

9. Harrison WG, Harris LR, & Irwin BD (1996) The kinetics of nitrogen utilization in the oceanic mixed layer: Nitrate and ammonium interactions at nanomolar concentrations. *Limnol. Oceanogr.* 41(1):16-32.

10. Sherwood TK, Pigford RL, & Wilke CR (1975) *Mass transfer* (McGraw-Hill Book Company, New York, USA).

11. Li YH & Gregory S (1974) Diffusion of ions in sea water and in deep-sea sediments. *Geochim. Cosmochim. Acta* 38(5):703-714.

12. Clift R, Grace JR, & Weber ME (1978) *Bubbles, drops, and particles* (Academic Press,, New York, London) p 380.

13. Svedén JB, Adam B, Walve J, Nahar N, Musat N, Lavik G *et al.* (2015) High cell-specific rates of nitrogen and carbon fixation by the cyanobacterium *Aphanizomenon* sp. at low temperatures in the Baltic Sea. *FEMS Microbiol. Ecol.* 91(12).

14. Verity PG, Robertson CY, Tronzo CR, Andrews MG, Nelson JR, & Sieracki ME (1992) Relationships between cell volume and the carbon and nitrogen content of marine photosynthetic nanoplankton. *Limnol. Oceanogr.* 37(7):1434-1446.

15. Olenina I, Hajdu S, Edler L, Andersson A, Wasmund N, Busch S *et al.* (2006) Biovolumes and size-classes of phytoplankton in the Baltic Sea. *HELCOM Balt. Sea Environ. Proc.* 106:144.

16. HELCOM (last updated July 2017) Manual for Marine Monitoring in the COMBINE Programme of HELCOM. (http://www.helcom.fi/action-areas/monitoring-and-assessment/manuals-and-guidelines/combine-manual).
